# Canonical and Noncanonical Autophagy as Potential Targets for COVID-19

**DOI:** 10.3390/cells9071619

**Published:** 2020-07-05

**Authors:** Melissa Bello-Perez, Isabel Sola, Beatriz Novoa, Daniel J. Klionsky, Alberto Falco

**Affiliations:** 1 Department of Molecular and Cell Biology, National Center of Biotechnology (CNB-CSIC), Campus Universidad Autónoma de Madrid, Darwin 3, 28049 Madrid, Spain; mloreto@cnb.csic.es (M.B.-P.); isola@cnb.csic.es (I.S.); 2 Institute of Marine Research (IIM), National Research Council (CSIC), 36208 Vigo, Spain; beatriznovoa@iim.csic.es; 3 Life Sciences Institute and Department of Molecular, Cellular and Developmental Biology, University of Michigan, Ann Arbor, MI 48109, USA; klionsky@umich.edu; 4 Institute of Research, Development, and Innovation in Healthcare Biotechnology in Elche (IDiBE), Miguel Hernández University (UMH), 03202 Elche, Spain

**Keywords:** antiviral, autophagy, canonical autophagy, coronavirus, COVID-19, noncanonical autophagy, SARS-CoV-2

## Abstract

The SARS-CoV-2 pandemic necessitates a review of the molecular mechanisms underlying cellular infection by coronaviruses, in order to identify potential therapeutic targets against the associated new disease (COVID-19). Previous studies on its counterparts prove a complex and concomitant interaction between coronaviruses and autophagy. The precise manipulation of this pathway allows these viruses to exploit the autophagy molecular machinery while avoiding its protective apoptotic drift and cellular innate immune responses. In turn, the maneuverability margins of such hijacking appear to be so narrow that the modulation of the autophagy, regardless of whether using inducers or inhibitors (many of which are FDA-approved for the treatment of other diseases), is usually detrimental to viral replication, including SARS-CoV-2. Recent discoveries indicate that these interactions stretch into the still poorly explored noncanonical autophagy pathway, which might play a substantial role in coronavirus replication. Still, some potential therapeutic targets within this pathway, such as RAB9 and its interacting proteins, look promising considering current knowledge. Thus, the combinatory treatment of COVID-19 with drugs affecting both canonical and noncanonical autophagy pathways may be a turning point in the fight against this and other viral infections, which may also imply beneficial prospects of long-term protection.

## 1. No Drug for this Bug (and Many Others) Yet

On 30 January 2020, the Emergency Committee of the World Health Organization (WHO) decreed the “Public Health Emergency of International Concern” (PHEIC) because of the outbreak of a coronavirus originated in the Chinese province of Hubei in late 2019, which has been called severe acute respiratory syndrome coronavirus 2 (SARS-CoV-2) [1] and is the causative agent of a deadly disease termed coronavirus disease 2019 (COVID-19). On 11 March, after confirming its spread across the world to hundreds of countries, the WHO announced it as a global pandemic.

The International Health Regulations 2005 (IHR 2005) [2] were signed by 194 states 23 May 2005 at the 58th World Health Assembly (entered into force on 15 June 2007). Since then, this is the sixth time WHO has decreed a PHEIC, and the second time ever that a pandemic is declared. If we include the epidemic of severe acute respiratory syndrome (SARS) disease caused by the SARS coronavirus (SARS-CoV) in 2002–2003, and which probably contributed to promoting the development of this regulation, there can be considered a total of seven PHEICs declared by WHO in less than 20 years, and all of them caused by viruses.

The general perspective is even more devastating if we add on top the incidence of other viral diseases including those already globally spread such as the seasonal flu, AIDS and hepatitis, as well as the recent spontaneous outbreaks of other emerging and considerably lethal, but less globally expanded diseases, such as the Middle East respiratory syndrome (MERS), also caused by a coronavirus (MERS-CoV), and dengue fever, caused by the dengue flavivirus (DENV). This scenario highlights the general lack of effective treatments against this growing number of emerging viral infections for which no vaccines are available, and whose morbidity and mortality rates are a major concern, even in developed countries, despite the availability of the ultimate technological and biomedical advances [3,4].

As it is currently happening, institutions mobilize resources towards the creation of vaccines against any outbreak; however, their development is not immediate. Material and human investments in the development of antivirals are, therefore, urgently needed to respond quickly when the infection bursts forth, but also to cope with subsequent outbreaks, whose intensity may remain high if no vaccination programs are executed, no herd immunity is generated or, in either case, the virus mutates sufficiently [4,5].

## 2. Studies on SARS-CoV-2’s Counterparts Might Reveal Therapeutic Targets

The ‘one bug, one drug’ approach to antiviral drug development, despite several successes and a catchy slogan, has proven inadequate for responding to situations such as the current one, which are remarkably numerous and dangerous. For the identification of antiviral therapeutic targets against pathogenic virus species, knowledge must be retrieved from published research concerning their closest viral family members.

SARS-CoV-2 belongs to the subfamily Orthocoronavirinae (order: Nidovirales; family: Coronaviridae) [1], commonly known as coronaviruses (Table 1). The single-stranded positive RNA genome of this group of enveloped viruses is 30 kb long, which is among the largest known RNA virus genomes. The 5’-terminal two-thirds of the genome include two overlapping open reading frames (ORFs), ORF1a and 1b, which encode 16 non-structural proteins (NSPs) involved in viral genome replication and subgenomic mRNA synthesis. The 3’-terminal third of the genome encodes four main structural proteins, i.e., spike (S), membrane (M), envelope (E) and nucleocapsid (N), and a number of genus-specific accessory proteins. These proteins are not essential for replication, but contribute to virulence mainly by modulating the innate immune response [6,7,8].

Virus species: HCoV-229E, human coronavirus 229E; HCoV-OC43, human coronavirus OC43; IBV, infectious bronchitis virus; MHV, murine hepatitis virus; PDCoV, porcine deltacoronavirus; PEDV, porcine epidemic diarrhea virus; TGEV, transmissible gastroenteritis virus.

Coronaviruses are classified into four genera based on genomic features: *Alphacoronavirus* and *Betacoronavirus*, which infect mammals (including humans), and *Gammacoronavirus* and *Deltacoronavirus*, which mostly infect birds, but also mammals. Among the five subgenera of *Betacoronavirus*, SARS-CoV-2 together with SARS-CoV belongs to the *Sarbecovirus* subgenus [1,5], which has been the subject of extensive study due to its high incidence and lethality in humans. Although SARS-CoV-2 is phylogenetically closer to some bat SARS-like coronaviruses [10,11], it shares approximately 80% of its genomic identity and homologous gene organization with SARS-CoV [6,11], which led to their classification within the same species, SARS-related coronavirus [1]. Therefore, to some extent, our knowledge on the former SARS-CoV may be applicable to this new virus.

The surface glycoprotein S mediates receptor binding and membrane fusion during the virus entry [7]. SARS-CoV and SARS-CoV-2 S proteins (hereafter termed S_cov_ and S_cov2_, respectively) share 76% amino acid sequence identity, which represents a high value considering it is one of the most exposed and thus variable proteins within the virus family [6,11,12]. Furthermore, such an identity is much higher in relevant functional regions because S_cov2_ conserves the typical elements necessary for its function: the receptor binding domain (RBD) in subunit 1 (S1), the fusion peptide (FP), the heptad repeats (HR) 1 and 2, the transmembrane domain (TM) and the cytoplasmic domain (CP) in S2 [6]. Additionally, despite minor differences, S_cov_ and S_cov2_ molecular structures, and their changes to adopt the fusion-competent conformation, are homologous, and 20 out of 22 N-glycosylations in S_cov2_ are conserved in S_cov_ [12,13]. In fact, early studies have reported that both S_cov_ and S_cov2_ (albeit S_cov2_ with a little more affinity [12,13]) recognize angiotensin-converting enzyme 2 (ACE2) as the cellular surface receptor that mediates the viral entry into the host [11,12,13]. Thus, in view of the high degree of homology shared with SARS-CoV, it is probable that SARS-CoV-2 may also enter cells by clathrin-dependent as well as by clathrin- and caveolae-independent endocytosis pathways, and then engage the endocytic pathway [14,15,16].

High ratios of protein sequence identity were also found for the 16 coronaviral NSPs between both SARS-CoVs. These values vary between 68 (NSP2) and 100% (NSP13), and half of them are over 95% (NSP5, 7-10 and 12-14). NSP6, which is widely addressed in the present work, shares an 88% identity between both viruses [6]. All NSPs play important roles in viral replication and transcription processes (for instance, NSP5, protease; NSP7, primase; NSP12, RNA-dependent RNA polymerase and NSP13, helicase). In particular, NSP6 is a transmembrane protein that complexes with NSP3 and NSP4 and is implicated in the formation of ER-derived double-membrane vesicles (DMVs) during coronavirus replication [17]. Such a high level of conservation in most NSPs from SARS-CoV and SARS-CoV-2 suggests close molecular structures and homologous functions, and thus parallel/comparable replication cycles.

Such observations are of great importance in helping to identify the molecular pathways interacting with and responding to a particular viral infection, because the previous acquired knowledge on their modulation might offer strategically important therapeutic advantages. Indeed, except for rare unknown or scarcely studied molecular pathways, an array of pharmacologically characterized drugs intended for modulating each route of interest are approved for treating certain diseases, and these can be expanded with corresponding repositioning.

## 3. Autophagy Interplays with the Replication Cycles of Multiple Virus Groups

**Figure 1:**
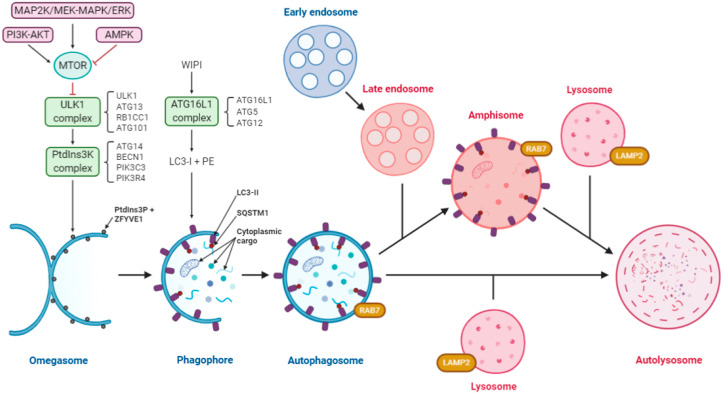
Diagram of the autophagy pathway including the convergence of the endocytic pathway. Autophagy is regulated by three protein complexes: ULK1, comprising of ULK1, ATG13, RB1CC1/FIP200 and ATG101; class III PtdIns3K, comprising of ATG14, BECN1, PIK3R4/VPS15 and PIK3C3/VPS34 and ATG16L1, comprising of ATG16L1, ATG5 and ATG12. Under starvation conditions, MTOR is inactivated allowing ULK1 complex formation, and activation of the PtdIns3K, which creates the PtdIns3P-rich regions on the surface of the omegasome. WIPI proteins recognize these domains and recruit the ATG16L1 complex, which facilitates lipidation of LC3-I to form LC3-II. Receptors such as SQSTM1/p62 bind to ubiquitinated cargo and LC3-II to facilitate selective autophagy. Cytoplasmic cargo includes damaged mitochondria, organelles, proteins, nucleic acids, intracellular bacteria, etc. Expansion of the phagophore through membrane addition sequesters a portion of the cytoplasm and upon closure forms the autophagosome. These autophagosomes are decorated with RAB7, which leads to the fusion with lysosomes to form the autolysosomes, where the cargo is degraded. The endocytic pathway (used by some viruses) and autophagy converge, resulting in the formation of an amphisome, which also fuses with lysosomes to form autolysosomes. The pink color indicates acidic compartments. Abbreviations: AMPK, AMP activated protein kinase; BCL2, BCL2 apoptosis regulator; BECN1, beclin 1; LAMP2, lysosomal associated membrane protein 2; MAP1LC3/LC3, microtubule associated protein 1 light chain 3; MTOR, mechanistic target of rapamycin kinase; PE, phosphoethanolamine; PIK3C3/VPS34, phosphatidylinositol 3-kinase catalytic subunit type 3; PIK3R4/VPS15, phosphoinositide-3-kinase regulatory subunit 4; PtdIns3K, phosphatidylinositol 3-kinase; Ptdins3P, phosphatidylinositol-3-phosphate; PTK2/FAK, protein tyrosine kinase 2; RAB7, RAB7, member RAS oncogene family; RB1CC1/FIP200, RB inducible coiled-coil 1; SQSTM1/p62, sequestosome 1; ULK1, unc-51 like autophagy activating kinase 1; WIPI1/2, WD repeat domain, phosphoinositide interacting 1/2; ZFYVE1/DFCP1, zinc finger FYVE-type containing 1.

Based on the literature, the macroautophagy (hereafter referred to as autophagy) pathway might be a promising target to tackle SARS-CoV-2 infection. Autophagy is a highly conserved eukaryotic process of cytoplasmic degradation that is activated, among others, under conditions of starvation and endoplasmic reticulum (ER) stress [18,19]. This mechanism maintains cellular homeostasis and requires the orchestration of a variety of molecules and dynamic membrane rearrangements to achieve complete autophagic flux. Briefly, in the canonical pathway of starvation-induced autophagy (see Figure 1 for a graphical description and abbreviations), the process begins with the generation of a sequestering compartment. This process involves the induction of an omega-shaped subdomain of the ER membrane (termed an omegasome) that evolves to form the phagophore. The latter is a transient structure, which consists of a double-membrane sheet that expands until it closes in on itself, wrapping recyclable cellular material and forming the autophagosome. Still bordered by a double membrane, the autophagosome may fuse with an acidic late endosome to form a single-membrane amphisome. Finally, the autophagosome or amphisome fuses with the lysosome to form the autolysosome, inside which the enzymatic degradation of the cargo occurs under acidic conditions. As expected, each phase of the process is supported and regulated by the sequential recruitment and action of numerous proteins (Figure 1), which are usually termed as autophagy related (ATG). The initiation of autophagy is regulated by the ULK1 and class III phosphatidylinositol 3-kinase (PtdIns3K) complexes (under the negative regulation of MTOR), which create domains that are enriched in the lipid PtdIns3P. These domains recruit various proteins including the proteolytically processed form of MAP1LC3/LC3 (termed LC3-I) and mediate its conjugation to the lipid phosphatidylethanolamine (to generate LC3-II). This recruitment is essential for phagophore closure, and therefore the formation of the autophagosome in the canonical autophagy pathway. A complex including ATG16L1 specifies the site of LC3 lipidation [18,19,20,21,22].

The autophagy process manages to also be selective through the use of specific receptors that link various ligands to the autophagic machinery through receptor binding to LC3 on the concave side of the phagophore. In the case of microbes, this is often achieved by ubiquitin-tagging the cargoes via the action of a group of E3 ligase-family proteins. Among other types of selective autophagy, xenophagy specifically targets intracellular pathogens for their degradation and further integration into both innate and adaptive immune responses [21,23,24,25,26]. Conversely, in response to this cell-protective autophagy, several different families of viruses, including coronavirus, have adapted by evolving a large variety of strategies to escape and/or to benefit via the inhibition and/or stimulation of autophagy at different stages of the process [23,27,28,29]. Thus, the identification of these interaction points might bring the opportunity to disrupt the viral replication cycle at specific stages by targeting selected steps of autophagy.

## 4. Both Autophagy and Coronavirus Induce the Formation of Analogous Vesicular Structures

The most apparent hint suggesting a connection between autophagy and the coronavirus replication cycle is the hallmark presence of DMVs in both cases [23,27,28]. Coronavirus induce characteristic rearrangements of the ER membranes towards the generation of connected DMVs and convoluted membranes. Altogether, this cytosolic reticulovesicular system works as a scaffold for viral RNA synthesis, and presumably protects the viral elements from the host’s defense mechanisms [17,30]. The precise functions and dynamics of these structures are not fully understood, but several viral NSPs have been implicated [30]. Among them, various in vitro studies highlight the crucial role of NSP6, one of the viral replicase proteins, to subvert the autophagic machinery for the generation of the DMVs [31]. Evidence shows that coronavirus NSP6s induce the formation of these membrane rearrangements from the ER [31,32]. So far, only SARS-CoV NSP6 has been reported to partially colocalize with released LC3-positive DMVs [31].

**Figure 2:**
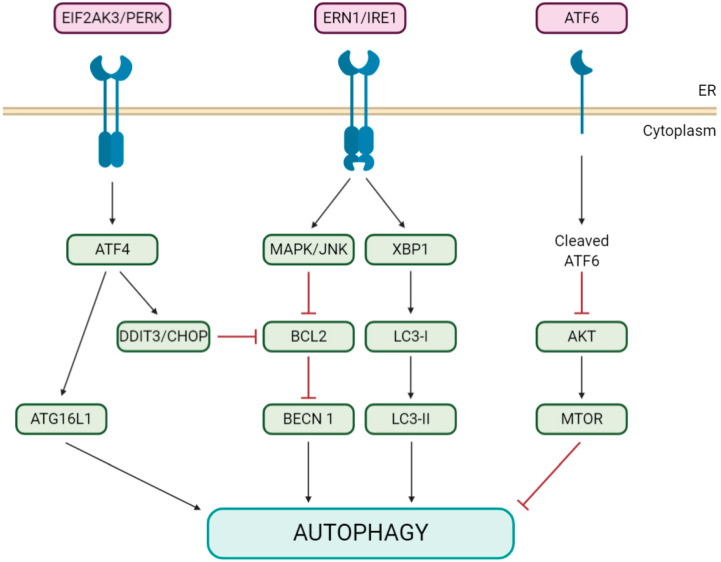
Diagram of the ER stress unfolded protein response (UPR) pathways triggering autophagy. ER stress can activate autophagy through three different UPR branches: EIF2AK3/PERK, ERN/IRE1 and/or the ATF6 signaling pathway. EIF2AK3/PERK induces autophagy by activating the ATG16L1 complex through ATF4 or by inducing DDIT3/CHOP expression, which indirectly causes BECN1 dissociation from BCL2. ERN/IRE1, through MAPK/JNK, mediates the phosphorylation of BCL2, which causes its dissociation from BECN1. The XBP1 branch enhances the formation of LC3-II. The ATF6 pathway also induces autophagy by inhibiting phosphorylation at the AKT-MTOR pathway. Abbreviations: AKT/PKB, AKT serine-threonine kinase; ATF4/6, activating transcription factor 4/6; DDIT3/CHOP/GADD153, DNA damage inducible transcript 3; EIF2AK3/PERK, eukaryotic translation initiation factor 2 alpha kinase 3; ERN/IRE1, endoplasmic reticulum to nucleus signaling 1; MAPK/JNK, mitogen-activated protein kinase and XBP1, X-box binding protein 1.

The initial mechanism underlying such activation remains unknown, although it has been shown for IBV NSP6 that it is not mediated by the inhibition of the signaling pathway of MTOR (the major autophagy suppressor [33]) [31], in contrast to a recent study on PEDV NSP6 [34]. Neither is it due to the activation of the NAD-dependent deacetylase SIRT1 (sirtuin 1; an MTOR-independent inducer of autophagy [35]) [31]. Alternatively, ER stress, which also triggers an autophagic process involving the unfolded protein response (UPR; see Figure 2 for a graphical description and abbreviations) [19,36], is induced by IBV infection via ERN1/IRE1 [37], one of three unfolded protein sensors in UPR signaling pathways, and a cellular autophagic response that has been reported as a prosurvival mechanism for ER stress [38]. Interestingly, just ERN1, but not its downstream effectors XBP1 and MAPK/JNK, is required for the induction of autophagy in IBV-infected cells, although its silencing does not inhibit IBV replication [39].

Regarding the other two known pathways of the UPR (Figure 2), their corresponding main regulators, ATF6 and EIF2AK3/PERK, are not modulated by IBV infection, and their silencing has no effect on IBV-induced autophagy [39]. In line with these results, Cottam et al. (2011) [31], also showed that IBV, MHV and SARS-CoV NSP6s do not exert any significant effect on either the activation of XBP1 from the IRE1 signaling pathway or the expression of the proapoptotic transcription factor DDIT3/CHOP/GADD153, from the EIF2AK3/PERK pathway.

## 5. Coronaviruses Appear to Modulate Pivotal Initiators of Both Autophagy and Apoptosis

In relation to the previously mentioned results, it is worth noting that the activation of the ERN1-MAPK signaling pathway in response to ER stress has been described to induce both autophagy [38] and cell death [40]. In this sense, despite the fact that the EIF2AK3/PERK pathway of the UPR appears to be irrelevant in coronavirus-induced autophagy, IBV induces apoptosis, and benefits from it, via the EIF2AK3/PERK and EIF2AK2/PKR activation of DDIT3/CHOP, which in turn suppresses the MAPK/ERK pathway [41]. This MAPK/ERK pathway together with the MAPK/JNK pathway, mediate noncanonical autophagy via the regulation of the BCL2-interacting protein BECN1 (beclin 1) [42].

Moreover, it has been reported that MERS-CoV cell culture infections induce substantial changes in the phosphorylation of relevant elements of not only the MAPK/ERK pathway (also known as the RAS-RAF-MAP2K/MEK-MAPK/ERK pathway), but also the class I phosphoinositide 3-kinase (PI3K)-AKT-MTOR pathway [43], which is also involved in apoptotic processes [40,44]. Remarkably, treatment with specific inhibitors of these two pathways and EIF2AK2/PKR, which have also been described to modulate autophagy, inhibit MERS-CoV infection [43]. Along these lines, it was also demonstrated recently that SKP2 (S-phase kinase associated protein 2), which is activated by AKT1, promotes BECN1 degradation and the inhibition of autophagy, and in turn that SKP2 suppression, and thus autophagy activation, inhibits MERS-CoV infection [45].

Within this context, some coronaviruses have evolved an additional mechanism to prevent apoptosis, as well as the host’s type I interferon (IFN) immune response, consisting of antagonizing the IFN-inducible OAS (2’-5’-oligoadenylate synthetase)-RNASEL (ribonuclease L) pathway and thus blocking RNASEL activity, i.e., the cleavage of viral and host single-stranded RNA, and subsequent cell death. To this end, the RNASEL activator 2’,5’-oligoadenylate is degraded by means of the cyclic phosphodiesterase activity of several betacoronaviral accessory proteins [46,47]. Paradoxically, it is described that RNASEL triggers autophagy in response to viral infections [48] via the MAPK/JNK pathway [49]; however, as mentioned previously, coronavirus already modulate autophagy by means of ERN1 with, apparently, no need of MAPK/JNK [39].

## 6. Coronavirus Corrupt and Block Autophagy via NSP6 and Some Accessory Proteins

Major controversy arises as to whether the LC3-containing vesicles induced during IBV [31,39,50], MHV [31,50,51], MERS-CoV [45], SARS-CoV [31,50] and PEDV [34] infections are actually autophagosomes. Such an effect has been demonstrated to be mediated by the viral NSP6s in some of these cases [31,34,41,50], and appears to be driven via an omegasome intermediate [31,50], similar to that seen in canonical starvation-induced autophagy [52]. Along this line, the generation of these coronavirus-induced autophagosomes requires the PtdIns3P-enrichment of the ER membrane outer leaflet, and the recruitment of ZFYVE1/DFCP1 (a key protein in omegasome formation), WIPI1/2, ATG5 and LC3-II (all components of the autophagic machinery), and SQSTM1/p62 (a receptor protein for selective autophagy) [31,50,51].

Conversely, the omegasomes, autophagosomes and autolysosomes in NSP6-expressing cells undergo a different maturation process from those induced by, for instance, just starvation. Cottam et al. (2014) [50] described that IBV, MHV and SARS-CoV NSP6s, and IBV infection, generate significantly smaller-diameter autophagosomes (Ø ≤ 0.5 µm) in comparison to the usual ones (Ø: about 1 µm). Further assays performed with IBV NSP6 in this regard show that its expression limits the expansion of both omegasomes and phagophores, even when they are induced by either starvation or inhibition of the MTOR kinase. In this same work, because they demonstrate that IBV NSP6 does not prevent the fusion of autophagosomes and lysosomes (as was also recently reported for IBV infection [39]), it is suggested that the reduced size of these autophagosomes limits their capacity to fuse with multiple lysosomes, generating smaller autolysosomes as a result. Interestingly, it is also shown that NSP6 inhibits the recruitment of MTOR to the surface of lysosomes, which may affect the activity of the final autolysosome.

A converging work recently reported that MERS-CoV infection actually blocks autophagy at the autolysosome formation stage via NSP6 and the accessory proteins 4b and 5. This finding is still consistent with previous data because, as a consequence, this block increases the total number of early-stage autophagic vesicles and reduces the autolysosome ratio therein [45], as it occurs even after treatment with the late autophagy blockers chloroquine or bafilomycin A_1_ [53,54,55]. Such an effect would benefit the viral replication in several ways: by preventing the maturation of endosomal and autophagic vesicles, and thus their potentially excessive degradative capacity with regard to viral elements at basically all the stages of their replication cycle, and providing them with all this new machinery for their replication in safe conditions [14,15,17,30,56].

## 7. Alternative Autophagy Pathways Might be Implicated in Coronavirus Infections

Consistent with this scenario, there are several supporting lines of evidence in which coronaviruses or their NSP6s (among others) induce not only the initiation of an autophagic process in the host, but also its blockade at a late stage of the process. For instance, there is the presence of common autophagy markers in vesicular structures from coronavirus infected- or NSP6-expressing cells (Table 2). The accumulation of LC3 and increased conversion to LC3-II [34,39,57,58], the accumulation of BECN1 [58] and the increased degradation of SQSTM1/p62 [57,58] also occurs. It is then not surprising that the reduction or abolition of these events when an essential autophagic element is blocked, such as the proteolytic cleavage, allows the conversion of LC3-I to LC3-II [31]. Neither is it when such elements are chemically inhibited, such as PIK3C3 with wortmannin to abolish the formation of the omegasome and thus the following autophagic processes such as LC3 recruitment and conversion [31,57]; or silenced, as seen for the example with *Atg5* [31,34,39].

However, the regulation of the canonical pathway of autophagy does not explain other wide-perspective observations, even considering the great differences that may exist among all the experimental systems used, i.e., different coronavirus genera/lineages and cell lines. The most contradictory fact, given the close interaction between coronaviral replication and autophagy pathways, is that the knockout of genes encoding autophagy-essential proteins such as *ATG5*, *ATG7*, *BECN1* or *LC3* di not abolish the replication in cell culture of the coronaviruses tested; actually, for many of them, replication was unaffected or increased (Table 3). The lack of these elements also does not prevent the generation of the ER pleiomorphic interconnected vesicular structures required for viral replication, although they were analyzed only in a few of these studies on MHV [51,59,61]. Another surprising finding is the only partial colocalization of autophagosomes (LC3-labelled in most studies) with viral replication elements that massively accumulate in infected cells (Table 2). In this sense, Snijder et al. (2006) [62] described complete separation of LC3 and SARS-CoV NSP3 subunits in Vero-E6 infected cells. All these facts together suggest an alternative autophagy pathway involved in coronavirus replication.

In this sense, Reggiori et al. (2010) [59] propose that coronaviruses induce ER-membrane rearrangements by manipulating the alternative ER-associated degradation pathway, specifically the selective clearing process of ER degradation enhancing alpha-mannosidase like protein 1 (EDEM1). EDEM1, probably together with other ER chaperones, is stored in vesicles termed EDEMosomes that are guided out of the ER by means of a COPII complex coat-independent mechanism, and delivered to endosomal compartments for disposal. In this work, it is suggested that MHV hijacks this pathway to promote the EDEM1-independent formation of viral DMVs coated with nonlipidated LC3 (LC3-I), which is essential for viral replication. By this approach, it made sense that ATG5 [31,39,45,51,57,58,60,61] and ATG7 [57,59], which are involved in LC3-I processing to LC3-II [59,61], are found dispensable for such a task. However, as discussed by the authors of the work, another route with these characteristics had just been revealed and might be implicated; ATG5- and ATG7-independent autophagy [63].

In 2009, Nishida et al. [63] described that certain autophagosomes may result from late endosomes and the trans-Golgi without participation of ATG5, ATG7 and LC3 conversion. Additionally, its chemical blockade by brefeldin A (BFA) suggests that the initial steps of this alternative autophagy pathway might involve the fusion of vesicles in the ER/cis-Golgi region [64], because this compound mediates the inhibition of ADP ribosylation factor 1 (ARF1), a GTPase from the RAS superfamily, which recruits coat proteins for the vesicular trafficking between both organelles [65]. Thus, ATG5- and ATG7-independent autophagy establishes an endocytic pathway-Golgi route able to potentially interact with the replication cycle of (mostly enveloped) viruses at multiple stages/processes: (1) their entry by endocytosis and fusion of viral and host membranes; (2) the processing of the viral glycoprotein carbohydrate moieties that requires their transfer from the ER to the cis-Golgi; (3) their intracellular transport; (4) replication; (5) assembly or (6) egress by means of host vesicular scaffolds.

As we know from many other viruses, not only the canonical but also the alternative autophagy pathways (or some of their elements) are hijacked and subverted for their replication [23,66], and BFA possesses antiviral activity in many cases [67,68,69,70], including coronaviruses [71]. For the elucidation of the particular contribution of each autophagy pathway to coronavirus replication cycles, further studies will be required including the use of modulators such as BFA together with the monounsaturated fatty acid oleate [64]; BFA does not affect canonical autophagy but it inhibits both the ATG5- and ATG7-independent [63,72] and the BECN1-independent process [73,74], whereas the BECN1-independent pathway is only induced by oleic acid [74]. Additionally, it will be necessary to add new members to the list of autophagy markers such as RAB9 for assessing the activity of the ATG5- and ATG7-independent pathway [63], which mediates the trafficking of late endosomes to the trans-Golgi [75].

## 8. Autophagy Modulators are Promising Anticoronavirals

The interplay between coronaviruses and autophagy is very complex and not completely understood. During a coronavirus infection, autophagy is both a cellular response mechanism and a viral replication tool. In fact, coronaviruses can both induce and inhibit autophagy with interactions at multiple levels within a narrow action area limited by apoptosis and the IFN response. Other representative examples of this complexity are that, although autophagy activation inhibits TGEV replication [57], a proviral mitochondria-selective autophagy is induced in TGEV-infected cells [76], or that PEDV induces autophagy and benefits from it [58], but it is also inhibited by rapamycin-induced autophagy [60].

For all this, and despite existing differences between studies that are almost certainly due to the use of distinct experimental systems, the modulation of autophagy usually affects the replication of coronaviruses, and therefore it becomes a promising therapeutic target in the search for anticoronavirals. Table 4 and Table 5 compile the reported effects of autophagy inducers or inhibitors, respectively, on the infection of different coronaviruses in cell cultures. Half of them are already FDA-approved drugs for other diseases/disorders, and several have already shown inhibitory activity against SARS-CoV-2, i.e., ivermectin, (hydroxy-) chloroquine and nitazoxanide. As can be observed, even classic modulators, such as rapamycin, 3-methyladenine (3-MA) or chloroquine, usually exert an effect on coronavirus replication. In general, among all the autophagy modulators tested, independently of being autophagy inducers or inhibitors, the outcome is usually antiviral activity. This fact may reflect not only the precise viral control over the autophagy pathway, but also the difficulty of maintaining such a balance and the detrimental effect on viral replication if there is any dysregulation in this back-and-forth game.

As shown in Table 4, autophagy inducers generally antagonize coronavirus replication. Among the autophagy inhibitors (Table 5), chloroquine (the most tested one) shows broad-spectrum anticoronaviral activity, which is probably because of its multimodal effects. Briefly, chloroquine, apart from disorganizing the Golgi, induces lysosomal alkalinization, which prevents amphisome/autophagosome-lysosome fusion and blocks the vesicle trafficking system [53,54,55,93], which potentially affects the replication cycle of coronavirus systemically, including their entry, which is mediated by pH-dependent endocytosis and requires a low pH for the S protein to trigger its membrane fusion activity [94,95]. Nitazoxanide is another late-stage autophagy blocker [96] that shows high anti-SARS-CoV-2 activity in cell cultures (IC_50_: 2.12 μM) [97], although it should be considered that its main metabolite, tizoxanide, induces autophagy by inhibiting the PI3K-AKT-MTOR pathway [98]. At this moment, the scientific community is focusing efforts in searching, by different approaches, for effective drugs against this pathogen and continuously revealing autophagy modulators [99,100,101].

**Table 5:** Effect of autophagy modulators on the replication levels of coronavirus in cell cultures.

Drug	Action Mechanism on Autophagy	Coronavirus Species	
		Inhibited	Non-Inhibited ^a^
3-MA	Inhibition of class III PtdIns3K [102]	MHV [51], PEDV [58]	
Bafilomycin A_1_	Inhibition of V-ATPase, raise lysosomal/vacuolar pH and inhibition of autolysosome formation [24,103]		PEDV [60]
(Hydroxy-) Chloroquine *	Raise lysosomal pH, inhibit autolysosome formation and disorganize Golgi [53]	PEDV [58], SARS-CoV [104,105] SARS-CoV-2 [97,106]	
GW5074/Dramafenib *	Inhibition of RAF1/c-Raf1 [107]	MERS-CoV [43]	
LY294002	Inhibitor of PtdIns3K and PI3K [108]		TGEV [57]
Nitazoxanide/Alinia *	Blockage of late-stage lysosome acidification [96]	SARS-CoV-2 [97]	
Reserpine *	Inhibitor of autolysosome formation [109]	SARS-CoV [79]	
UO126	Inhibition of MAPK/ERK pathway [73]	MERS-CoV [43]	
Wortmannin	Inhibitor of PtdIns3K and PI3Ks [108]	MERS-CoV [43]	IBV [31], PEDV [60], TGEV [57]

^a^ non-affected or increased. * FDA approved drugs. In bold, drugs showing IC_50_ ≤ 1 µM. Cell lines used: Huh7 [43] for MERS-CoV; IPEC-J2 [60] for PEDV; MEF [51] for MHV; ST [57] for TGEV; Vero for IBV [31] and SARS-CoV-2 [106]; Vero-E6 for PEDV [58], SARS-CoV [79,104,105] and SARS-CoV-2 [97].

## 9. Outlook and Challenges

As shown here, drugs that target autophagy, as well as those involved in regulating the endocytic pathway [110] could be added to the arsenal of compounds against coronavirus infections (for an extensive list see Zumla et al. (2016) [111]). As a consequence, the discovery of new autophagy regulatory drugs may be a source of new antivirals that is worth testing for this purpose. In this sense, we propose that the alternative autophagy routes are still scarcely explored in this field and can provide unexpected positive outcomes in the fight against viruses, and particularly coronaviruses. In a follow-up prospective effort, we think that interference with RAB9 activity, a key element in these pathways, might be a promising approach. In this sense, the targeting of GDI/RabGDI (GDP dissociation inhibitor), which forms a complex with RAB9 in the cytosol and mediates its activity in the endosome-trans Golgi network, and specific “GDI-displacement factors” such as RABAC1/Yip3 (Rab acceptor 1) are also candidates worth testing for this purpose [112,113].

To conclude, we observed that most of the reviewed works tested the anticoronaviral effect of each autophagy modulator individually in order to accurately unravel the mechanisms involved. Thus, having shown that autophagy and coronavirus replication cycles converge in several different stages, treatment strategies including the combination of autophagy-modulating agents might result in synergistic effects that are worth studying. In this vein, among present combinatory treatments, a frequent one is (hydroxy-) chloroquine together with azithromycin [114,115], a macrolide antibiotic with extensively reported autophagy-blocking activity, as well as other family members [116,117]. Another important factor to consider is the scarce number of in vivo studies in this field [118], which is certainly due to the required and necessary biosafety restrictions. However, these studies are essential to assess the true potential of these drugs for clinical implementation because the outcome within the complex biosystem can be very different from that of in-cell culture tests. In this sense, despite the inhibitory effects observed in vitro, (hydroxy-) chloroquine treatments, either alone or in combination with azithromycin, has shown no benefits against SARS-CoV-2 infection in clinical trials [119,120]. Besides, the in vivo context allows the identification of not only possible side effects, but also paradoxical issues such as the fact that the virulence of coronaviruses may be different even if showing similar replication levels [8]. Finally, it is important to mention that autophagy also plays a significant role in adaptive immune responses [24,25,26], and in vivo tests are essential for determining the possible implications in this sense when using autophagic modulators in experimental treatments, as they could be either detrimental or beneficial in the long term.

## Figures and Tables

**Table 1:** Classification of the subfamily Orthocoronavirinae [1,5,9].

Genus (No. Subgenera)	Subgenus ^a^	Species ^b^	Disease and Host
*Alphacoronavirus* (14)	*Duvinacovirus*	HCoV-229E	Common cold in humans
	*Tegacovirus*	TGEV	Transmissible gastroenteritis disease in pigs
	*Pedacovirus*	PEDV	Porcine epidemic diarrhea disease
*Betacoronavirus* (5)	*Embecovirus*	HCoV-OC43 MHV	Common cold in humans Murine hepatitis disease
	*Merbecovirus*	MERS-CoV	MERS in humans
	*Sarbecovirus*	SARS-CoV* SARS-CoV-2*	SARS in humans COVID-19 in humans
*Deltacoronavirus* (3)	*Buldecovirus*	PDCoV	Acute gastrointestinal disorders in neonatal piglets
*Gammacoronavirus* (3)	*Igacovirus*	IBV	Infectious bronchitis disease in chickens

^a^ the table only lists subgenera for species presented in the table. ^b^ species selected because of their relevance in humans and human activity and/or research. * both viruses belong to the same SARS-CoV-related species [1].

**Table 2:** Autophagy markers and colocalizing viral elements detected in vesicles induced by coronavirus.

Virus/NSP6	Cell Lines	Autophagy Vesicle Marker	Colocalized Viral Protein/Element
IBV	Vero	LC3	dsRNA [31]
		WIPI2, ATG5 [31]	
MHV	HEK293	LC3	NSP2/3 [59]
	HeLa	LC3	NSP2/3 [59]
	MEF	LC3	N, p22, Hel, M [51], NSP2/3 [59]
		ATG12	N [51]
PEDV	Vero-E6	LC3 [58]	
	IPEC-J2	LC3	N [60]
SARS-CoV	Vero	LC3	Replicase proteins [61]
TGEV	ST	LC3 [57]	
IBV NSP6	CHO	LC3, SQSTM1/p62 [31]	
	HEK293	ATG5, ZFYVE1/DFCP1 [31]	
	MEF	LC3 [31]	
	Vero	LC3, WIPI2 [50]	
MHV NSP6	CHO	LC3 [31]	
	Vero	LC3 [50]	
SARS-CoV NSP6	CHO	LC3	NSP6 [31]

Cell lines: CHO, Chinese hamster ovary cells; HEK293, human embryonic kidney 293 cells; HeLa, human cervix adenocarcinoma epithelial cells; IPEC-J2, intestinal porcine epithelial cells; MEF, mouse embryonic fibroblast; ST, pig testis fibroblast cells; Vero, kidney epithelial cells from African green monkey.

**Table 3:** Effect of the silencing of autophagy-essential elements on coronavirus replication.

Gene ^a^	Levels of Viral Infection/Replication ^b^		
	Lower	Equal	Higher
*ATG5*/*Atg5*	MHV [51], PEDV [58]	IBV [31,39], MHV [61]	MERS-CoV [45], TGEV [57]
*ATG7*/*Atg7*		MHV [59]	TGEV [57]
*BECN1*/*Becn1*	PEDV [58]	IBV [39]	
*LC3*/*Lc3*	MHV [59]		TGEV [57]

^a^ silenced or knocked out gene (human/mouse). ^b^ in comparison to non-infected cells. Cell lines used: Vero [31] and human non-small cell lung carcinoma cells (H1299) [39] for IBV; Vero-B4 [45] for MERS-CoV; MEF [51,59,61] and mouse macrophages [61] for MHV; Vero-E6 [58] for PEDV and ST [57] for TGEV.

**Table 4:** Effect of autophagy inducers on the replication levels of coronavirus in cell cultures.

Drug	Action Mechanism on Autophagy	Coronavirus Species	
		Inhibited	Non-Inhibited ^a^
ABT-737/Venetoclax *	Release of BECN1 from BCL2 and BCL2L1/Bcl-X_L_ interaction [77]	MERS-CoV [45]	
Aescim	Activation of ROS-MAPK/p38 signaling pathway [78]	SARS-CoV [79]	
Everolimus/Afinitor *	Inhibition of MTOR [80]	MERS-CoV [43]	
GF109203X	Inhibition of PRKC/PKC (protein kinase C) [81]	MERS-CoV [43]	
Ivermectin *	Inhibition of PAK1 and subsequent AKT phosphorylation [82]	SARS-CoV-2 [83]	
Niclosamide *	Inhibition of MTORC1 and ULK1 activities and induction of LC3B expression [83,84]	MERS-CoV [45]	
Rapamycin/ Sirolumus *	Inhibition of MTOR [33]	MERS-CoV [43], MHV [59], TGEV [57], PEDV [60]	PEDV [58]
Ro-31-8220	Inhibition of PRKC/PKC [85,86]	MERS-CoV [43]	
Selumetinib *	Inhibitor of MAP2K1/MEK1-MAP2K2/MEK2 [87]	MERS-CoV [43]	
SMIP004	Inhibition of SKP2 [88]	MERS-CoV [45]	
Sorafenib/Nexavar *	Inhibition of RAF-MAP2K-MAPK/ERK signaling pathway and VEGF receptor tyrosine kinase [89] and activation of AKT [90]	MERS-CoV [43]	
Trametinib/Tafinlar *	Inhibitor of MAP2K1/MEK1-MAP2K2/MEK2 [91]	MERS-CoV [43]	
Valinomycin	Electrogenic K^+^ ionophore that causes loss of the mitochondrial membrane potential and stimulates mitophagy [92]	MERS-CoV [45], SARS-CoV [79]	

^a^ non-affected or increased. * FDA approved drugs. In bold, drugs showing IC_50_ ≤ 1 µM. Cell lines used: Huh7 [43] and Vero-B4 [45] for MERS-CoV; IPEC-J2 [60] for PEDV; MEF [59] for MHV; ST [57] for TGEV; Vero/hSLAM for SARS-CoV-2 [83], Vero-E6 for PEDV [58] and SARS-CoV [79].
